# Successes and Short Comings in Four Years of an International External Quality Assurance Program for Animal Influenza Surveillance

**DOI:** 10.1371/journal.pone.0164261

**Published:** 2016-10-27

**Authors:** Erica Spackman, Carol Cardona, Jeannette Muñoz-Aguayo, Susan Fleming

**Affiliations:** 1 Exotic and Emerging Avian Viral Disease Unit, Southeast Poultry Research Laboratory, US National Poultry Research Center, USDA-Agricultural Research Service, Athens, GA, United States of America; 2 Department of Veterinary and Biomedical Sciences, University of Minnesota, St. Paul, MN, United States of America; Food and Drug Administration, UNITED STATES

## Abstract

The US National institutes of Health-Centers of Excellence for Influenza Research and Surveillance is a research consortium that funds numerous labs worldwide to conduct influenza A surveillance in diverse animal species. There is no harmonization of testing procedures among these labs; therefore an external quality assurance (EQA) program was implemented to evaluate testing accuracy among labs in the program in 2012. Accurate detection of novel influenza A variants is crucial because of the broad host range and potentially high virulence of the virus in diverse species. Two molecular detection sample sets and 2 serology sample sets (one with avian origin isolates, and one with mammalian origin isolates each) were made available at approximately six month intervals. Participating labs tested the material in accordance with their own protocols. During a five year period a total of 41 labs from 23 countries ordered a total of 132 avian molecular, 121 mammalian molecular and 90 serology sample sets. Testing was completed by 111 individuals. Detection of type A influenza by RT-PCR was reliable with a pass rate (80% or greater agreement with expected results) of 86.6% for avian and 86.2% for mammalian origin isolates. However, identification of subtype by RT-PCR was relatively poor with 54.1% and 75.9% accuracy for avian and mammalian influenza isolates respectively. Serological testing had an overall pass rate of 86.9% and 22/23 labs used commercial ELISA kits. Based on the results of this EQA program six labs modified their procedures to improve accuracy and one lab identified an unknown equipment problem. These data represent the successful implementation of an international EQA program for an infectious disease; insights into the logistics and test design are also discussed.

## Introduction

The US National Institutes of Health (NIH) funded five multi-institutional research and surveillance centers for influenza (Centers of Excellent for Influenza Research and Surveillance [CEIRS]) starting in 2006. One of the two primary focus areas of the CEIRS program is to conduct influenza surveillance in domestic and wild animals with the aim of identifying novel and emergent influenza A strains which could transmit to humans.

The structure of the CEIRS surveillance labs is somewhat unique. Unlike most networks of government or academically affiliated veterinary diagnostic or public health labs, there are no specific recommendations or standards provided for influenza A testing or for influenza A antibody detection. Individual labs select the most appropriate test for their workflow and specimen type. Furthermore, the diversity of influenza these labs could encounter is unusually broad. First, the labs may test specimens from potentially any animal species although wild waterfowl, domestic poultry, swine, sea mammals and horses are among the most common target species. Secondly, the surveillance labs are located world-wide in 23 countries and include labs on each of the 6 inhabited continents; therefore influenza A from any geographic lineage may be present in samples.

In 2012 an external quality assurance (EQA) program was implemented for the CEIRS animal surveillance labs based on the framework described by Wiegers [1]. Similar to other EQA programs [2] the goal was to ensure that all participating labs were utilizing tests with adequate sensitivity and specificity and to provide a way for labs to evaluate and train their personnel for adequate performance. Here we report the results of 5 years of testing and discuss the implementation of an international EQA testing for an infectious disease.

## Methods

### General overview of testing and logistics

Two sample types were distributed for molecular testing for influenza A and optional subtype identification: 1) avian origin influenza virus, and 2) mammalian origin influenza virus. A third sample set consisted of animal origin serum to evaluate detection of antibodies to influenza A and alternated between swine and chicken sera. Each lab selected which sample sets they would complete. Testing was conducted eight times at approximately six month intervals from June 2012 through February 2016. Labs were only required to participate once per year, but could participate more often.

The materials were distributed in coded vials labeled with sample set date, sample number and lot number. Labs requiring more than one set of any type were provided with different lots (each lot had a unique composition and sample order) to minimize the influence from one individual’s results on another individual’s interpretation. Samples remained blinded until all results had been returned. Labs were instructed to process the material exactly as they would process their routine surveillance samples.

For reporting purposes 80% or greater correlation with the expected results was considered “passing”. A cut-off of 80% for a passing score was selected because this is the value used by the Clinical Laboratories Improvement Amendments for proficiency testing in clinical laboratories for virological tests of moderate and high complexity [3].

Although Ct values for rRT-PCR, or OD readings for serology, were requested on score sheets, scoring was based on how the technician interpreted the result, i.e. as positive or negative. This approach was implemented because the goal of testing was to imitate a clinical sample and the practical question was whether a sample would be treated as positive or negative for follow-up and eventual reporting by the individual conducting the test. Importantly, due to variations in testing methods and training of individuals there was no standard for cut-off values by Ct value and OD readings (and not all labs used tests which had numerical result). Labs with technicians that scored below 80% on any test were contacted to identify the possible cause. Subtype identification was optional and was only scored for samples and subtypes for which the lab tested. For example, if a lab tested an H3N2 for H3, but not N2, they would only be scored for whether they identified H3 correctly. A result was treated as a false negative if the actual subtype was tested for, but not identified and if no other subtype was identified in the sample (i.e. it was negative by all attempted tests). A result was considered a false positive when another subtype was incorrectly identified, regardless of whether a test for the actual subtype was conducted.

### Molecular sample sets

The molecular detection sample sets were comprised of inactivated whole virus. Only isolates not classified as “select agents” by the US Department of Agriculture (USDA) were utilized. A limited number of strains that had been modified to be non-pathogenic by reverse genetics (and which had removed from the select agent list by USDA) were included because of the importance of these strains for subtype identification. Influenza A was propagated in embryonated chickens eggs (ECE) or cell culture by standard methods as appropriate for each isolate. Virus was inactivated in 1% Environ (Steris Inc. Mentor, OH) in distilled water, which has been shown to stabilize the RNA [4] for the first four rounds of testing or 0.1% beta-propiolactone in brain heart infusion broth for the last four rounds of testing [5]. Material for each sample was safety tested by completing two replicates of two blind passages each, in ECE using standard methods for influenza [6] to ensure that the virus had been completely inactivated. Each isolate was then diluted to a pre-determined target concentration and the concentration was confirmed by testing in triplicate to ensure that the target concentration was reliably achieved.

Each sample set included a total of 10 samples which represented a range of concentrations: one to three negative (no template) samples and the remaining samples were a mixture of weak positive, positive and strong positive concentrations. The Ct value ranges were based on the ranges of mean egg infectious dose (EID_50_) equivalents observed for virus shed by infected animals using primers and probe that are a100% match to the virus sequence (e.g. the strong positive was approximately equivalent to 10^5^−10^7^ EID_50_ per ml with a Ct value of <26.9; a positive was approximately equivalent to 10^3^−10^5^ EID_50_ per ml with a Ct value range of 27.0–32.9; and weak positives were approximately equivalent to 10^1^−10^3^ EID_50_ per ml for Ct values ≥33). Negative samples contained no template and had an expected Ct value of 0.0. Isolates were selected to represent numerous genetic lineages of the influenza M gene from around the world. Because the M gene was the only gene targeted by the type A influenza RT-PCR tests utilized by participating labs diversity in this gene was important to demonstrate adequate sensitivity with variants. Additional criteria for isolate selection were inclusion of the HA and NA subtypes and lineages that would be most relevant for the surveillance target populations.

### Internal quality control and assurance of molecular samples

Initial preparation of the sample sets included testing all materials at their final target concentration in triplicate to ensure that the target concentration was achieved and that reproducibility was adequate. Avian molecular samples were internally calibrated with two different real-time PCR instruments each with RT-PCR reagents from a different manufacturer (7500 FAST [Applied Biosystems, Foster City, CA] with the Ambion AgPath kit [Life Technologies, Waltham, MA] and the Smart Cycler II [Cepheid, Inc., Carlsbad, CA] with the OneStep RT-PCR kit [Qiagen, Inc, Valencia, CA]). Both variations used the USDA M gene primers and probes [7]. After the second round of testing it was determined that use of a second test did not provide better information and was discontinued; only the 7500 FAST based test was used subsequently. Mammalian molecular samples were only tested with the 7500 FAST and Ambion AgPath kit, using USDA M gene primers and probes [7].

The test materials were monitored for deterioration by running material that was retained at 1–2 week intervals until all results were returned. Molecular sample sets were stored at both the recommended 4°C and at -20°C, ambient temperature and 37°C to evaluate the effect of different temperatures on sample stability.

### Serology sample material

Each serology sample set consisted of 15 samples for the first two rounds, then 10 samples in subsequent rounds of testing. One or two negative samples were included in each set and the remaining samples were adjusted to represent a range of antibody concentrations (weak positive through strong positive) with sterile phosphate buffered saline. All sera were treated at 56°C for one hour in a water bath prior to internal characterization. Diluted sera were tested every two weeks by the methods listed below to ensure sample stability. Mammalian and avian sample sets were initially offered simultaneously, by the third round of testing the species of origin alternated between avian and swine to simplify logistics.

To produce sera for the avian antibody sample set, isolates were selected for diversity in the NP protein sequence because this protein is targeted by most in-house and commercial type A influenza antibody tests. The isolates, in allantoic fluid, were inactivated with 0.1% beta-propiolactone and were used to prepare a vaccine with a commercial oil adjuvant, Montanide ISA 70VG (Seppic, Inc. Fairfield., NJ), in accordance with the manufacturer’s instructions. Five week-old specific pathogen free white leghorn chickens were then vaccinated with 0.5ml per bird by the subcutaneous route. Serum was collected 3-weeks later and tested by commercial avian influenza ELISA kits (IDEXX, Westbrook, ME; and Biochek, Scarborough, ME). This specific serum generation in chickens was approved by the Southeast Poultry Research Lab, Institutional Animal Care and Use committee. Serum was only tested by agar gel immunodiffusion (AGID) by standard methods [8] if results were returned from labs which used AGID.

Convalescent positive and negative swine sera were obtained from pigs experimentally infected with swine influenza virus (National Center for Animal Diseases, USDA; using IACUC approved procedures) or from commercial pigs in the US. Each serum sample was tested by commercial blocking-ELISA (bELISA) (MultiS-Screen, IDEXX, Westbrook, ME) to characterize the antibody concentration.

## Results

### Logistics

Attempts were made to minimize transit time; the goal was less than 48hrs in transit and to ensure that materials would pass through customs. Therefore it was critical to contact the recipients prior to shipment and acquire all permits in advance. The most common requirement were letters stating methods of testing, certification that safety testing was completed, and that all material was non-infectious. One country required isolate-by-isolate safety testing data, which is only available a short time prior to shipment, and because of the length of the permitting process, the permits were not issued before the sample material had expired and the material could not be shipped. Two other countries which did not require the same in depth data also had lengthy permitting processes which prevented shipment. Most countries had no permit requirements since the material was non-infectious, although certification letter were required for customs clearance.

In most cases there were no issues with clearing customs. However, one country to which six shipments were completed, had very inconsistent requirements and the length of time the material would spend waiting to clear was variable and, in one shipment expired as it spent more than six weeks in customs. Further complicating shipment was that in some cases the recipient could obtain permits for the serology samples, but not molecular samples and vice versa.

### Participation

During the eight rounds of testing, 41 labs (a lab is defined as groups under a single principal investigator) from 23 countries ordered at total of 132 avian influenza molecular sample sets, 121 mammalian influenza molecular sample sets and 90 serology sample sets (Table 1). Results were returned from: 119 (90.1%) avian molecular, 109 (90.1%) mammalian molecular, and 69 (76.6%) serology sample sets.

**Table 1:** Summary of external quality assurance sample ordering, completion and results returned.

	Avian influenza molecular	Mammalian influenza molecular	Serology
Total ordered	132	121	90
Results returned	119	109	69
Total participating labs	31	21	23
Total individuals participating	71	56	45
Percent returned	90.1%	90.1%	76.6%
Percent pass (≥80%)	86.6%	86.2%	87.0%

Of the total tests ordered, results were not returned or the EQA testing could not be completed for 13.5% (46/343) of all sets of samples ordered. Reasons included: 1) import permits could not be obtained, so the material could not be shipped; 2) shipments were delayed in transit or customs and the samples degraded over time or from high temperatures so could not be used; 3) there were technical or logistical problems at the labs, e.g. moving, new personnel, equipment or reagent problems, the lab was tasked to work on an emergency outbreak situation; 4) the participating lab never provided results and never responded to inquiries; and 5) labs returned results for fewer sample sets than they received.

A total of 111 individual technicians completed at least one type of EQA tests in at least one round of testing. Of the 111 technicians, 31 completed two types of samples and 15 completed sample sets for all the types (avian, mammalian and serology). Participants also held diverse roles in the lab: principal investigators, lab managers, professional technicians, post-docs, graduate students and temporary technicians.

### Avian influenza molecular samples

A total of 119 avian influenza molecular sample sets were completed for influenza A detection by 71 technicians from 31 labs. Technicians passed by scoring 80% or greater agreement with expected results on 103 (86.6%) of the samples sets (Table 1). The discrepant results consisted of 18.2% false positives and 81.8% false negatives.

Twenty-eight labs reported methods data. The RNA extraction procedures, RT-PCR reagents, primers and probe sequences and instruments varied widely among the participants. All labs reported using commercial RNA extraction kits, however even labs that used the same kits, sometimes used different procedures based on differing starting volumes and final RNA volumes. Real-time RT-PCR (rRT-PCR) was the most common platform and was used by 26 of the 28 labs that reported methods; the remaining two labs used conventional RT-PCR. All rRT-PCR and RT-PCR based tests targeted the M gene and the most common primer/probe sets were the USDA avian influenza test [7] (8 labs used as reported or with modifications), the US CDC/WHO universal fluA primers [9] (used by 5 labs) or the Fouchier primers [10] (used by 3 labs). The remaining labs reported using other published or non-published primers and probes. Three labs did not provide information on the specific primers sets utilized. The RT-PCR reagents were highly variable among labs regardless of the primers and probe sets utilized and were dictated by regional availability and lab preference.

Subtype testing was optional and was completed by technicians from 16 labs. Individual labs tested for different subtypes including: H1 including both 2009 pandemic (pdm(H1N1)09) specific tests and non-pdm(H1N1)09 H1 tests, and, H3, H5, H6, H7, H9, N1 and N2 in samples. Subtype identification was correct for 100 (54.1%) of the subtype tests run (n = 185). False negatives (the test for the correct subtype was negative) accounted for 46.3% of the discrepant results and false positives (an assay was positive for the wrong subtype) accounted for 53.7% of the discrepant results (Table 2).

**Table 2:** Accuracy of subtype identification by RT-PCR (conventional and real-time).

Test type	Correct/total ID (percent correct)	Percent false positive	Percent false negative
Avian	100/185(54.1%)	53.7	46.3
Mammalian	155/204(76.0%)	20.4	79.6

Based on poor results during the first two rounds of testing two labs changed their M gene primers and probes from an in-house set to a published set and two labs added or modified their H5 or H7 subtype tests to expand testing specificity. The changes resulted in improvements in all subsequent rounds of testing in which they participated.

### Mammalian influenza molecular samples

A total of 109 mammalian influenza molecular sample sets were completed for influenza A detection by 56 technicians from 21 labs. Passing scores were achieved on a total of 94 (86.2%) sample sets (Table 1). Discrepant results were comprised of 29.0% false positives and 71.0% false negatives.

Methods data were reported by 19 of 22 labs. All labs used tests which targeted the M gene, however one technician reported using a test targeting the NP gene, although the other technicians in the same lab used an M gene based test. One lab used conventional RT-PCR; all others used real-time RT-PCR tests. Similar to the avian molecular testing, most labs used the USDA type A influenza test or modifications of it (8 labs) [7, 11], the US CDC/WHO primers [9] (5 labs) or the Fouchier test [10] (2 labs). The remaining labs used other published or in-house primers and probes. Similar to the avian samples, RT-PCR reagents were highly variable among labs participating in mammalian influenza testing.

Subtype identification was attempted by 12 labs with either real-time RT-PCR or conventional RT-PCR. The actual subtypes assayed varied among labs but included: H1 (both non-pdm(H1N1)09 and pdm(H1N1)09), H3, H5, N1 and N2. Overall the correct subtype was identified by 155 of 204 tests (76.0% correct). Incorrect results included 20.4% false positives and 79.6% false negatives (Table 2).

Two labs modified their type A influenza testing procedures to improve sensitivity with a more diverse set of isolates. One participated in subsequent rounds of testing and improved their score.

### General molecular testing results

Fifteen labs participated in testing with both the avian and mammalian molecular sample sets, of which 10 used the same procedure for both sets of samples (seven labs used the USDA test or modified USDA test and three labs used CDC/WHO M gene primers for both avian and mammalian testing, one used an in-house test). Four labs used different primers for avian and mammalian samples. No labs reported using an internal positive control.

Twenty-five technicians from 10 of the 35 labs that conducted testing with molecular methods for type A influenza (avian or mammalian) received a failing score (less than 80% agreement with expected results) at some time. Thirty-three percent of the failing scores were from one lab. Six of those labs completed avian and mammalian samples sets and poor scores were observed with both; the remaining four labs only participated in the avian EQA. Nine of these technicians participated in at least one more round of testing and six improved their scores. Reasons for failure were determined by following-up with the lab and included: the participant was inexperienced with the technique, a reagent failure occurred, sample degradation from spending an extended time (greater than six weeks) in customs, or the RT-PCR procedure was insensitive and required modification.

### Serology

A total of 69 serology samples sets for influenza A antibody were completed by 45 individual technicians from 23 labs. Passing scores were achieved with 60 (87.0%) of the sample sets. Commercial ELISA kits were utilized by 19 labs (13 of which were from one manufacturer), one lab used AGID (three total tests) and three labs did not report serological methods. Eleven labs used ELISA kits that were not species specific (i.e. blocking ELISA format). Discrepant ELISA results consisted of 98% false negatives and 2% false positives and 100% of the AGID discrepant results were false negatives.

Nine technicians from four labs did not pass a serology test at some time during the EQA program. Reasons for failure were traced back to inexperienced technicians, use of AGID, or use of a new ELISA kit.

### General results and outcomes

Data integrity errors were noted with 16 sets of results, all from research labs. Typical problems were that the wrong lot or no lot number was reported, the wrong result reporting sheet was used, the results sheet was altered, or data were missing. Testing also helped to identify an equipment problem in one lab. Three labs used the EQA testing material to train new personnel or to help verify new or updated procedures. Another positive outcome was that four labs that did not have access to a reliable source of positive control material retained samples for future use as control material.

## Discussion

An EQA program was implemented for surveillance labs in year five of the CEIRS program with the goals of: ensuring accurate, consistent results; detecting problems with procedures or operator errors; and to provide material which could be used for assay verification or technician training. Surveillance labs in the CEIRS network are located worldwide and test specimens from diverse domestic and wild avian and mammalian species with the goal of identifying and characterizing novel influenza A viruses in animals. Therefore, the full diversity of influenza A could be present in samples processed by these labs; it is critical for labs to detect unusual isolates (either novel lineages or lineages which have been disseminated from their normal host or geographic region). The labs themselves are also functionally diverse, and included: research labs, veterinary diagnostic labs and even two labs that processed human samples in addition to the animal surveillance samples. Participating labs were government, academic and private, which accounts for the diversity in roles among the participants (students through principal investigators). Interestingly, data integrity issues were exclusively with research labs and may reflect a difference in training, where diagnostic labs place more emphasis on data handling. Participation at the individual level also indicated that most technicians are specialized between the molecular and serological tests; only 25.5% (28/111) participated in both serological and molecular testing.

Detection of influenza A (avian or mammalian origin) by molecular methods was very reliable despite a lack of harmonization of the testing procedures. Adequate personnel training and utilization of properly validated methods were crucial. Type A influenza detection discrepancies were mostly false negatives, which suggest sensitivity was the problem more often than cross-contamination (or sample mix-ups). Importantly, the discrepant samples were not always the weakest samples based on analyte concentration. The sensitivity of individual tests correlated to the match between primer/probe sequences and the sample sequence. Similarly, the performance characteristics of the tests varied substantially among the isolates as evidenced by large variations in Ct values for individual samples, for example strong positives could have high Ct values when the primers/probe were a poor sequence match for the isolate (data not shown). Wide variations in Ct values were also reported by Popowich *et al*. [12]. In general it would be advisable for labs to use influenza detection assays that are well validated and which are continually monitored for performance with emerging lineages (e.g. tests which are used by national and international diagnostic laboratory networks).

Subtype identification was poor for both avian (54.1% accuracy) and mammalian (75.9% accuracy) isolates. The slightly better accuracy with mammalian isolates is likely due to there being fewer subtypes and less variability among the mammalian lineages, as the isolates are predominantly H1, H3, N1 and N2. Importantly both false negatives and false positives accounted for the errors. False negatives were due to mismatches between the primers and probes. False positives were due to cross reactions, since primers and probes often targeted conserved regions, which are sometimes adequately conserved enough between subtypes for binding to occur. Importantly, subtype identification was attempted by only a few labs. The consequence of misidentified subtypes in this context is unclear since most of the labs will confirm the subtype by sequencing and/or serology. Because of the poor accuracy of identifying random subtypes in avian surveillance samples by rRT-PCR, laboratories should consider using gene sequencing instead.

The accuracy of the molecular testing was overall slightly lower than results reported from other influenza molecular proficiency testing of US public health labs, international veterinary diagnostic labs and Chinese public health labs [4, 12, 13]. The difference is probably due to the wider diversity of influenza A isolates used in this testing versus the other testing programs which focused on few or even a single lineage. Testing with a variety of isolates is critical for animal influenza surveillance labs since they can encounter the broadest diversity of isolates relative to public health or regional veterinary diagnostic labs. In contrast Stelzer-Braid reported poorer results with H5N1 testing, but attributed that to the inclusion of very weak samples as results improved when labs modified testing to optimize their limits of detection [14].

Antibody detection was highly reliable with commercial ELISA kits. The few false positives could be attributed to variations in instruments used to read the plates, how well samples were mixed, how the plates were washed, and how long the plates were left before reading (extended times could result in a negative control with an optical density that is too high and a reduced ability to detect true positives). Agar gel immuno-diffusion was utilized by only a few labs, but was prone to false negatives. Compared to commercial ELISA, interpreting AGID requires more skill and the reference reagents must be carefully calibrated to achieve adequate sensitivity. Commercial ELISA kits may be more expensive than AGID on a per sample basis, but assuming they undergo some form of licensure, they offer consistent quality control of the kit materials from the manufacturer, robustness, and a simple format where the results can usually be interpreted by software.

One feature of real samples that the molecular EQA samples could not reproduce was the sample matrices that each lab could encounter. These were clean samples, without inhibitors, so the robustness of RNA extraction procedures could not be evaluated. Utilization of an internal positive control or extraction positive control would help ensure that negative results are reliable. Similarly, the serological samples were from only a few species and variations in test sensitivity based on species variations could not be determined. Variation in the sensitivity of blocking ELISAs for antibody from different species has been documented [15–17].

Overall, the EQA testing had an appreciable impact on the methods and approaches of participating labs. Six labs provided feedback that they modified their molecular detection procedure to improve detection or subtype identification based on the results of the EQA testing. Five of those labs participated in subsequent rounds of testing and improved their scores. Another lab identified an equipment problem when poor results were observed. They were able to fix the problem and complete a repeat of the test within the same testing cycle and achieve 100% agreement with expected results. Several labs were also able to use the material to train technicians and verify their proficiency or to verify new test methods. Importantly, the poor results for influenza A detection or antibody detection were accounted for by only a few labs, indicating that training may be needed since the reported test procedures were validated and should have been adequate. Finally, the results observed here demonstrate a lack of reliability for influenza A subtype identification by RT-PCR methods.
